# Applications of telemedicine in the supply and distribution of COVID-19 vaccines in Africa

**DOI:** 10.7189/jogh.11.03039

**Published:** 2021-03-01

**Authors:** Melody Okereke, Abdulhammed Opeyemi Babatunde, Samson Tobiloba Samuel, Isaac Olushola Ogunkola, Yidnekachew Girma Mogessie, Don Eliseo Lucero-Prisno

**Affiliations:** 1 Faculty of Pharmaceutical Sciences, University of Ilorin, Kwara State, Nigeria; 2 Medicine and Surgery, Faculty of Clinical Sciences, College of Medicine, University of Ibadan, Ibadan, Nigeria; 3 Faculty of Dentistry, College of Medicine, University of Ibadan, Ibadan, Nigeria; 4 Department of Public Health sciences, University of Calabar, Calabar, Nigeria; 5 St. Paul's Hospital Millennium Medical College, Addis Ababa, Ethiopia; 6 Department of Global Health and Development, London School of Hygiene and Tropical Medicine, UK

The urgency for the COVID-19 vaccine became pertinent globally as the virus continued to threaten global health and economies. Scientists around the world were charged with the responsibility to develop a safe and effective COVID-19 vaccine. About 50 out of over 300 different vaccine candidates are undergoing clinical trials and funded by organizations such as the Global Alliance for Vaccines and Immunizations (GAVI), Coalition for Epidemic Preparedness Innovations (CEPI), and World Health Organization [1]. In early December, the world recorded a significant milestone in the quest for COVID-19 vaccine development as the Pfizer-BioNTech COVID-19 vaccine was approved after completion of phase III trial [2]. The approval of this vaccine generated optimism for the subsequent approval of other vaccine candidates currently in phase III trial. Therefore, governments need to employ innovative strategies to administer the COVID-19 vaccines while also adhering to personal preventive measures. These may include the use of telemedicine. Telemedicine, a practice that uses telecommunications networks to provide health care, which has been adopted in mass vaccinations during previous disease outbreaks [3] has the potential of providing promising outcomes in course of the projected COVID-19 mass vaccinations in Africa. However, these are not without challenges. Generally, the prominent challenges of telemedicine in Africa include low literacy level, especially in the rural areas, relatively high cost of internet, inadequate power supply, data privacy [4], cultural misconceptions and misinformation [5]. COVID-19 vaccination requires universal coverage whereas most telemedicine services are concentrated in urban settings due to the aforementioned challenges which are greatest in rural areas. Hence, there may be low community acceptance of telemedicine approaches for COVID-19 vaccination in Africa. There is a need for context-specific telemedicine projects in different locations and proper community engagement during the development and implementation stages to enhance uptake and acceptability.

**Figure:**
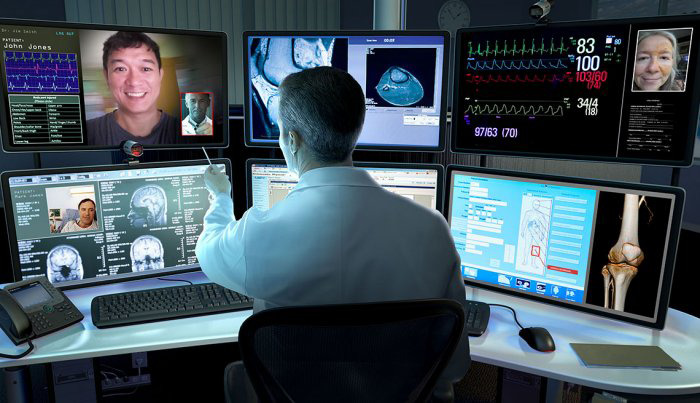
Photo: Via https://www.aarp.org/health/conditions-treatments/info-2018/telemedicine-teleheath-online-doctors-appointment.html (used with permission).

Telemedicine is a significant advancement in health care delivery in the 21st century that allows access to health information and services across distance through information and communication technology (ICT). Different telemedicine projects have been successfully implemented in Africa, especially mobile health technology (mHealth) through downloadable mobile applications, text messages, websites, and USSD code [6]. Growing penetration of smartphones and internet connection across African countries provides an opportunity for mHealth for health awareness, drug supply and management, disease surveillance and interventions, and virtual consultation with health professionals globally [6]. As African countries also prepare to commence vaccination, there is a need to employ a fast, efficient, and cost-effective strategy for the supply and administration of the COVID-19 vaccine. Hence, it is noteworthy to highlight the several opportunities that telemedicine provides in the course of the COVID-19 mass vaccination.

## POSSIBILITIES OF TELEMEDICINE IN COVID-19 MASS VACCINATION IN AFRICA

Although telemedicine has been in existence since the 1960s and over two decades ago in Africa [6], with the advent of COVID-19, it offers a viable alternative to the traditional means of health consultation. Telemedicine offers mildly ill patients an opportunity to access the supportive medical care they need while also minimizing their exposure to acutely ill patients. Telemedicine has been adopted in the mapping, distribution, and follow up of people during previous vaccination campaigns. For instance, the increasing penetration of mobile technology, which has reached about 95% of the world population's habitation played a major role in boosting efforts geared towards polio eradication [3]. As the access to mobile technology increases, its use in m-Health to enhance immunization campaign also increased. Through M-Health, authorities gathered real-time data, used geospatial mapping for enhancing surveillance, and monitored mobile populations through cellular phone data [3]. M-Health was also adopted as a tool to influence vaccine acceptance and build confidence. This immediate access to information by people may influence their decision to partake in vaccination. Also, M-Health using text messaging was found to be effective for behavioral changes towards Human papillomavirus (HPV) vaccination among the high-risk population for cervical cancer in the United States [7]. M-Screening intervention significantly increased knowledge, intent to receive, and receipt of HPV vaccines [7]. This approach which helped to lower the burden of human papillomavirus and cervical cancer in the United States [7] can as well provide promising potentials for the ongoing COVID-19 mass vaccination in Africa.

## PRE-VACCINATION CAMPAIGNS AND TELEMEDICINE

With the discovery of the COVID-19 vaccine and the peculiarities of the coronavirus, countries across the world are already deploying technology through telemedicine to safely acquire, distribute, and follow up the vaccination process [1]. For instance, Jordan, the United States, Saudi Arabia, amongst other countries have developed websites and mobile applications for citizens to register for COVID-19 vaccines [1]. These websites allow individuals to check for eligibility, schedule appointments, and identify inoculation centers whilst adhering to precautionary measures- without close contact and over-crowding. These approaches are worth adopting by African countries to ensure a hitch-free pre-vaccination campaign.

## VACCINATION, FOLLOW-UP AND TELEMEDICINE

The role of effective and timely communication in an ongoing COVID-19 mass vaccination cannot be overemphasized. For vaccination against COVID-19, there is a need for double and more robust efforts. Globally, there are concerns about the effectiveness of the vaccine, the speed of the development, and its safety [1]. To build confidence and encourage vaccination, as already in use by the World Health Organization and several countries of the world, the deployment of telemedicine in the form of artificial intelligence (AI) on the dissemination of COVID-19 information has been playing a critical role [8] towards addressing COVID-19 misinformation which is widespread in African countries [5]. The use of an AI-powered conversational chatbot could be leveraged. Towards enhancing COVID-19 mass vaccination via a telemedicine approach, accurate and updated information on the COVID-19 vaccine, close-by vaccination centers, and scheduling of vaccination could be easily accessed by people through the use of an AI-powered conversational chatbot [8]. Also, the COVID-19 vaccine developed by Pfizer in partnership with the German drug manufacturer, BioNTech requires storage at a temperature of minus 70°C [1] while the one developed by Moderna also requires freezing at the temperature of minus 20°C [1]. These are challenging conditions that are increasingly difficult to meet, especially in Africa where most countries have a poor and inadequate power supply. To mitigate wastage of the vaccine, strengthen supply chain networks and ensure extensive coverage of the vaccine, telemedicine could be adopted through data analysis tools provided by the approved COVID-19 teams or taskforce. In Nigeria, the use of data on the NCDC website to draw insights could aid the effective distribution of vaccine to regions with the highest incidence and people at risk. Furthermore, drones can be used for real-time delivery of vaccines to hard-to-reach communities. This is essential to improving access to health care which was disrupted by the COVID-19 pandemic in Africa [9].

## POST-VACCINATION AND TELEMEDICINE

Through Electronic Medical Record (EMR), another form of telemedicine, people who have been vaccinated could be monitored. This may be carried out through the implementation of an automated vaccine adverse effect surveillance and reporting system that is based on ambulatory electronic medical records [10]. This automated system would easily flag the potential adverse effects of the vaccine and help authorities promptly respond. It would also raise people's confidence in the vaccination as they are sure of close surveillance, proper monitoring and prompt response in the event of a negative reaction to the vaccine.

## RECOMMENDATION AND CONCLUSION

Although telemedicine coverage in Africa is still limited compared to the developed world due to the lack of political commitment, poor infrastructure, and inadequate resources, it has promising potentials to facilitate prompt access to the COVID-19 vaccine, routine follow-up post-vaccination, and surveillance in Africa. A simple user-friendly website or mobile application can be created for citizens to register for vaccination, have location mapped out for door-step vaccination, conduct routine assessment online in case of adverse drug reaction, and also store quality data for the vaccination program. Besides, text messages and USSD codes in African local languages can be adopted for awareness and registration for vaccination in areas with poor internet facility. Moreover, vaccinators can be provided with smartphones to input data of vaccinated areas and individuals to evaluate coverage and assist in policy-making for other vaccination programs. Although telemedicine cannot directly replace the traditional physical vaccination process, it can facilitate access in hard-to-reach areas and monitor the supply and distribution of COVID-19 vaccine to the end-users.
